# Multigene methylation analysis of enriched circulating tumor cells associates with poor progression-free survival in metastatic breast cancer patients

**DOI:** 10.18632/oncotarget.21426

**Published:** 2017-09-30

**Authors:** Theresa Benezeder, Verena Tiran, Alexandra A.N. Treitler, Christoph Suppan, Christopher Rossmann, Herbert Stoeger, Richard J. Cote, Ram H. Datar, Marija Balic, Nadia Dandachi

**Affiliations:** ^1^ Medical University of Graz, Department of Internal Medicine, Division of Oncology, Graz, Austria; ^2^ University of Miami Miller School of Medicine, Department of Pathology, Miami, Florida, U.S.A; ^3^ Medical University of Graz, Research Unit Circulating Tumor Cells and Cancer Stem Cells, Graz, Austria; ^4^ Medical University of Graz, Research Unit Epigenetic and Genetic Cancer Biomarkers, Division of Oncology, Graz, Austria

**Keywords:** circulating tumor cells, enrichment, metastatic breast cancer, methylation, prognosis

## Abstract

Blood-based biomarkers such as circulating tumor cells (CTCs) provide dynamic real-time assessment of molecular tumor characteristics beyond the primary tumor. The aim of this study was to evaluate the feasibility of a size-based microfilter to assess multigene methylation analysis of enriched CTCs in a prospective proof-of principle study. We examined the quantitative methylation status of nine genes (*AKR1B1, BMP6, CST6, HOXB4, HIST1H3C, ITIH5, NEUROD1, RASSF1, SOX17*) in enriched CTCs from metastatic breast cancer patients. Feasibility and clinical performance testing were assessed in a test set consisting of 37 patients and 25 healthy controls. With established cut-off values from the healthy control group, methylation of enriched CTCs was detected in at least one gene in 18/37 patients (48.6%), while 97.8% of all control samples were unmethylated. Patients with CTCs unmethylated for *CST6*, *ITIH5*, or *RASSF1* showed significantly longer PFS compared to patients with corresponding enriched methylated CTCs. This proof-of-principle study shows the feasibility of a size-based microfilter to enrich and analyze multigene methylation profile of CTCs from metastatic breast cancer patients. For the first time, we report that multigene methylation analysis of enriched CTCs provides prognostic information in metastatic breast cancer patients.

## INTRODUCTION

Breast cancer is the most frequently diagnosed cancer and the leading cause of cancer death among females worldwide, accounting for 25% of total cancer cases and 15% of all cancer deaths among females [1]. While chances of being cured of breast cancer have increased over the last decade, metastatic breast cancer remains essentially incurable and accounts for the majority of disease-related mortality [2]. Clearly, new prognostic and predictive biomarkers are needed in the metastatic setting. In this context, minimal-invasive blood-based biomarkers are a valuable source, providing important clinical and biological information of the tumor. In particular, blood-based biomarkers allow a real-time assessment of the molecular tumor genotype, thereby offering the possibility to monitor temporal and clonal evolution of tumor cells beyond the primary tumor.

Tumor cells that have left the primary tumor or metastases can be found in the circulation of patients and these circulating tumor cells (CTCs) can provide unique biological and clinical information [3]. Several studies have clearly documented the prognostic value of CTCs in a number of solid tumor malignancies [4–9], and these blood-based biomarkers have been acknowledged as a liquid biopsy allowing the monitoring of disease progression and efficacy of cancer treatment [3]. Most of these studies have used the affinity-based CellSearch system (Janssen Diagnostics, NJ, USA), which is also the only technology approved by the US Food and Drug Administration for CTC enumeration [9–11]. However, an important and well-known limitation of the CellSearch platform is that enrichment is based on tumor cells expressing epithelial cell adhesion molecules (EpCAM). Subpopulations of cells that have low or absent EpCAM expression may be missed by this system. Importantly, acquisition of a mesenchymal phenotype has been shown to be a frequent characteristic of epithelial cancer cells during disease progression and metastasis [12] and this process is also accompanied by down-regulation of epithelial markers such as EpCAM [13–15].

Due to the low number of CTCs in blood, even in metastatic patients, detection and molecular characterization of CTCs remain challenging. To address these difficulties, a variety of techniques has been used for enrichment and detection of CTCs using cell surface markers or morphological differences between CTCs and normal blood cells [16, 17]. In this project, we used an enrichment technique that captures CTCs based on their size and is independent of EpCAM expression on CTCs [18, 19].

With the increasing numbers of CTC studies, it has also become clear that beyond enumeration of CTCs, molecular characterization is necessary to better understand the role of CTCs in tumor progression and metastasis [3, 20, 21]. Importantly, a recent study indicates that CTCs are highly heterogeneous even within the same patient [22]. Taken together, studying CTCs has considerable potential to improve our current understanding of tumor heterogeneity and metastatic disease.

Altered DNA methylation is a well-established and frequent molecular hallmark of cancer cells. Particularly, hypermethylation of specific genes leads to gene silencing and contributes to the malignant phenotype [23]. Several genes have already been shown to be hypermethylated in breast cancer and some methylation profiles have also been associated with distinct breast cancer subtypes [24]. DNA methylation also occurs in genes involved in epithelial-mesenchymal-transition (EMT) [24], a process associated with tumor cell dissemination and metastasis. In most tumors, DNA methylation alterations are more frequent than genetic mutations, offering a wide range of potential targets [25].

So far, only few studies exist on DNA methylation of CTCs, but they already provide evidence for a potential biological and clinical role [26–29]. These studies mainly used antibody-based enrichment of CTCs and no study so far has evaluated size-based enrichment technologies in association with CTC methylation. Considering the accumulated evidence for the role of methylation in cancer progression and metastasis, further studies on the epigenetics of CTCs are definitively needed.

In this project, we used a size-based microfilter to enrich for CTCs with subsequent multigene methylation analysis of captured CTCs. We examined promoter methylation of nine candidate genes that are commonly methylated in breast cancer (*AKR1B1, BMP6, CST6, HOXB4, HIST1H3C, ITIH5, NEUROD1, RASSF1, SOX17*) [26, 30–32]. To accurately quantify DNA methylation, we used pyrosequencing, which offers a sensitive and highly reproducible method [33].

## RESULTS

### CTC enumeration

Using our size-based microfilter, 37 samples were tested for the presence of CTCs identified as nucleated CK+/DAPI+/CD45- cells. 19 out of 37 patients (51.4%) had >= 1 CTC per 7.5ml blood, with a median of 1 cell (range 0-56 cells). 18 patients (48.6%) had no CTCs, 10 patients (27.1%) had 1-4 CTCs and another 9 patients (24.3%) had >= 5 CTCs/7.5ml blood.

### Analytical performance of pyrosequencing assays

#### Linearity, reproducibility, and limit of detection (LOD)

We assessed the analytical performance of our pyrosequencing assays using a series of control samples. First, in order to test the linearity of the pyrosequencing assays, we generated dilution series consisting of unmethylated and fully methylated control samples at various percentages of methylation (0%, 5%, 10%, 25%, 50%, 75% and 100%). We found a strong linear correlation between theoretical and observed methylation values for each gene as well as for the calculated CMI for all nine genes with R^2^ values ranging from 0.961 to 0.997, demonstrating that there is no amplification bias and methylated and unmethylated molecules are equally amplified. The generated dilution series were also used to calculate the limit of detection (LOD) for all nine assays according to Armbruster et al [34]. Calculated LOD values ranged from 1.1% to 4.1%, limits generally reported for pyrosequencing assays [35]. As part of the quality control and in order to assess the robustness and reproducibility of pyrosequencing, we analyzed results from the same set of bisulfite-modified controls (MCF7, H1299 and MNCs) tested on different days in different pyrosequencing runs. The median of the gene-specific coefficient of variation (CV) value for the methylated control samples (MCF7 and H1299) was 2.1% (range 0.5% to 4.2%), and for the unmethylated control sample (MNCs) 10.1 (range 8.7% and 26.7%). These data demonstrate a high analytical performance of all pyrosequencing assays with reproducible run-to-run variations of control samples allowing for accurate and reproducible quantification of DNA methylation.

#### Assay validation using spiked-in breast cancer cells

**Figure 1:**
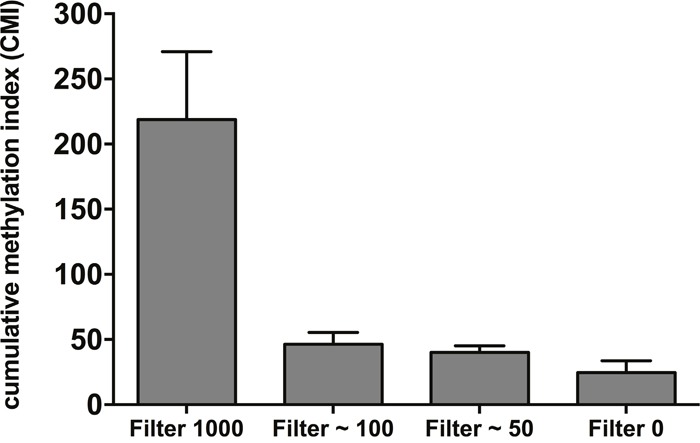
A cumulative methylation index (CMI) was calculated as the sum of the methylation percentage for the genes analyzed by spiking increasing numbers of MCF7 cells in 7.5ml healthy blood. CMI values were obtained from four independent spiking experiments and data are represented as mean ± SD

Next, blood samples from healthy controls were spiked with varying amounts of MCF7 (50-1000) cells and blood was then subjected to CTC enrichment with the size-based microfilter. Considering the analytical LOD of pyrosequencing (in general between 1 and 5%), the lowest number of spiked cells was 50 cells in 7.5ml blood (corresponding to about 6 cells per ml blood). After CTC capture, DNA was extracted, bisulfite-converted and tested by pyrosequencing for the 9-gene panel, excluding *HIST1H3C*, which is not methylated in MCF7 cells. Calculated CMI values were higher in all samples spiked with MCF7 cells compared to the unspiked sample (Figure 1), demonstrating the technical feasibility of the size-based microfilter for multigene methylation analysis.

### Clinical performance of multigene CTC methylation analysis

Finally, to verify the performance of our 9-gene panel for detecting methylation status of CTCs, we evaluated a clinical test set consisting of metastatic breast cancer patients (n=37) and healthy controls (n=25). Supplementary Table 2 summarizes all methylation results as well as CTC counts for each individual patient.

First, we assessed DNA methylation of all nine candidate genes in MNCs isolated from 25 healthy controls, representing the major source of DNA remaining on the microfilter. Quantitative methylation data from this control group was used to establish a cut-off for positive methylation according to Lehmann et al [36]. Table 1 shows the mean methylation values for all genes in the control group and the corresponding calculated cut-off values. These cut-off values were all higher than the calculated analytical LODs. Based on these cut-off values, *AKR1B1*, *NEUROD1* and *SOX17* were each methylated in 1/25 (4.0%) control samples, *HIST1H3C* methylation was observed in 2/25 (8.0%) control samples. All other genes (*BMP6, CST6, HOXB4, ITIH5*, and *RASSF1*) were unmethylated in the control group. Overall, 97.8% of all control samples were unmethylated.

**Table 1:** DNA methylation levels in peripheral blood cells from healthy controls (n=25) and corresponding cut-off values

Genes	Mean methylation level	SD	Cut-off^*^
*AKR1B1*	2.5	0.8	4.2
*BMP6*	3.9	1.1	6.1
*CST6*	5.1	0.9	6.9
*HIST1H3C*	4.7	1.8	8.3
*HOXB4*	2.4	0.7	3.8
*ITIH5*	4.7	1.1	6.8
*NEUROD1*	5.3	0.9	7.1
*RASSF1*	3.1	1.2	5.6
*SOX17*	9.7	2.6	15.0

^*^ Cut-off defined as mean + 2x SD [35].

**Figure 2:**
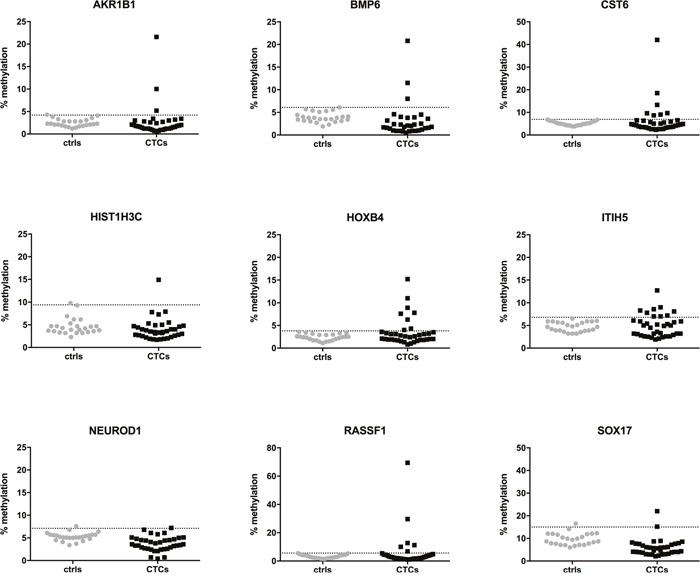
Quantitative methylation percentages of the 9 candidate genes analyzed by pyrosequencing in CTCs from metastatic breast cancer patients (n=37, black dots) and MNCs from healthy controls (n=25, grey dots) The dotted horizontal line represents the cut-off for positive methylation.

**Figure 3:**
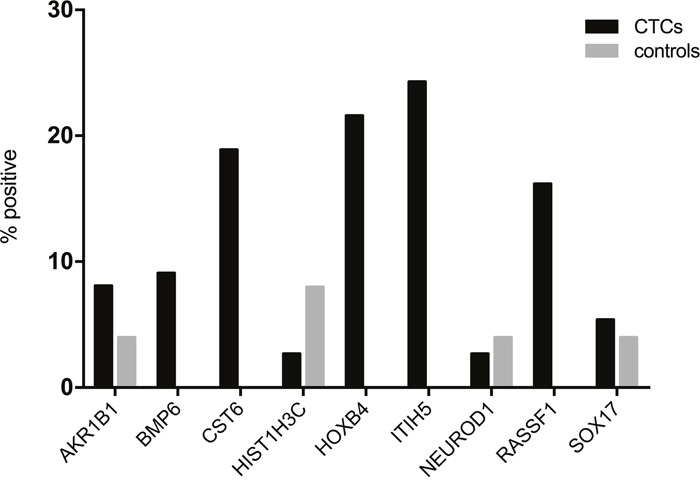
*CST6, HOXB4, ITIH5* and *RASSF1* are more frequently methylated in CTCs from metastatic breast cancer patients (black bars) compared to MNCs from healthy controls (grey bars) Samples were defined as positive when the mean methylation value for a sample was higher than the calculated cut-off value for the same gene.

Next, we assessed the methylation status of enriched CTCs from metastatic breast cancer patients. Quantitative methylation results for each gene are shown in Figure 2. Using the established cut-off values from the healthy control group, methylation of CTCs was detected in at least one gene in 18/37 patients (48.6%). Of these positive samples, at least two genes were methylated in 61.1% of patients (11/18), while only one gene was methylated in seven patients (38.9%). Among all genes tested, *ITIH5* (9/37) and *HOXB4* (8/37) were most frequently methylated in enriched CTCs, followed by *CST6* (7/37) and *RASSF1* (6/37). *AKR1B1* was methylated in 3/37 patients, *SOX17* in 2/37 patients, and *HIST1H3C* and *NEUROD1* were each only methylated in one of the 37 patients tested. PCR amplification failed for the *BMP6* gene in four patients despite repeating amplification twice, and was methylated in three of the 33 remaining patients. Overall, *CST6*, *HOXB4*, *ITIH5* and *RASSF1* were more frequently methylated in CTCs from breast cancer patients compared to MNCs from healthy controls (Figure 3).

There was no association between positive CTC count and frequency of methylated genes in the CTC enriched cell fraction (p = 0.330, Fisher's exact test). In detail, out of 19 patients with >= 1 CTC, 8 patients were unmethylated in all genes tested and 11 patients were methylated in at least one gene. On the other hand, out of 18 patients with no detectable CTCs, 11 patients showed no methylation and seven patients had at least one gene methylated. These results suggest that heterogeneous subpopulations of CTCs exist and that different subpopulations of CTCs may be identified using methylation profiling and CTC enumeration.

### Association between CTC methylation and clinicopathologic features

Next, the methylation status of candidate genes was associated with known clinicopathologic characteristics, including age at blood sampling, tumor grade, tumor size, node status, ER, PR status, tumor subtype, location of metastases and number of metastatic sites. We found no significant associations between CTC methylation and hormone receptor status, HER2 status, node status, tumor subtype, number of metastatic sites or age (data not shown). Patients without liver metastasis had a significantly higher proportion of unmethylated *HOXB4* compared to patients with liver metastasis (Fisher's Exact Test, p = 0.021). Additionally, patients with bone metastases were predominantly unmethylated with *ITIH5* compared to patients without bone metastases (Fisher's Exact Test, p = 0.042). All other genes were not significantly associated with location of metastasis. Taken together, these results indicate that frequency of CTC methylation was not associated with classical clinical features of metastatic breast cancer patients in our study cohort.

### Clinical utility of CTC methylation analysis

Finally, we evaluated the prognostic value of the most frequently methylated genes including *CST6, HOXB4, ITIH5* and *RASSF1*, in enriched CTCs from 32 metastatic breast cancer patients. During the follow-up period 6 of 32 patients died and 18 of 32 patients progressed. The median follow-up time for the patients still alive at the end of the study was 338 days ± 6.3. In our study group, negative ER status and high tumor grade were associated with poor PFS in univariate analysis (data not shown). None of the classical prognostic parameters were associated with OS. This lack of significance for OS might be explained by the short follow-up time with a low number of death events (6/32 patients) in this study. Therefore, in subsequent univariate analysis we only focused on PFS.

**Figure 4:**
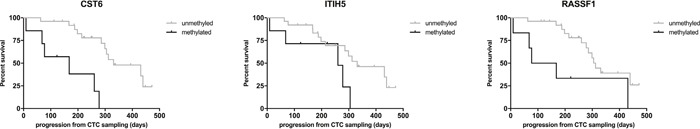
Impact of CTC methylation on progression-free survival (PFS) in metastatic breast cancer patients PFS for patients with *CST6, ITIH5*, and *RASSF1* methylation. black line, CTCs methylated, grey line, CTCs unmethylated.

Methylation of *CST6, ITIH5* and *RASSF1* in enriched CTCs were significantly associated with shorter PFS in metastatic breast cancer patients. As shown in Figure 4, patients with methylated enriched CTCs, showed disease progression with a median PFS time of 168 days (*CST6*), 260 days (*ITIH5*), and 77 days (*RASSF1*). In comparison, patients without CTC methylation demonstrated disease progression with a median PFS time of 331 days (*CST6* and *ITIH5*), 312 days (*RASSF1*). Compared to patients without methylation, patients with enriched CTC methylation had a significantly higher risk of disease progression (Table 2). In contrast, methylation of *HOXB4* was not significantly associated with PFS. Interestingly, CTC count was also not associated with PFS in our study group. CTC methylation of these genes also predicted shorter progression-free survival in the subgroup of patients with HR+/HER2+ phenotype (n=20) (data not shown).

**Table 2:** Univariate Cox regression analysis of PFS

Variable	n	HR (95% CI)	P value
*CST6* methylation			
Negative	25	1.00	
Positive	7	7.99 (2.33-26.84)	< 0.0001
*ITIH5* methylation			
Negative	25	1.00	
Positive	7	2.93 (0.97-8.88)	0.058
*RASSF1* methylation			
Negative	26	1.00	
Positive	6	3.17 (1.10-9.17)	0.033

HR hazard ratio, CI confidence interval.

Taken together, our data indicate that multigene methylation analysis of CTCs associates with poor PFS and thus may be useful in identifying patients at high risk for disease progression.

## DISCUSSION

In this proof-of principle study, we could successfully demonstrate the feasibility of analyzing multigene methylation profiles of size-based enriched CTCs from patients with metastatic breast cancer. We used quantitative pyrosequencing to identify the methylation status of nine marker genes that have previously shown to be hypermethylated in breast cancer patients. We also provide evidence for the potential clinical value of CTC multigene methylation analysis, demonstrating an association between methylated enriched CTCs and poor progression-free survival.

Methylation alterations are established hallmarks in tumors and are more frequent than genetic mutations, providing a wide range of possible targets [23, 25]. So far, only few studies exist on CTC methylation, but they highlight first biological and clinical evidence of CTC methylation. Chimonidou and colleagues demonstrated in three studies that methylation status of three tumor-associated genes (*CST6*, *BRMS1*, and *SOX17*) can be detected in circulating tumor cells of breast cancer patients [26, 28, 29]. However, limitations of these studies were the use of semi-quantitative MSP and only a limited number of genes were analyzed.

To our knowledge, this is the first study to use a size-based enrichment platform and pyrosequencing to successfully assess multigene methylation profiling of CTC enriched cell fractions. The performance of the size-based microfilter to capture CTCs with a high efficiency has already been published elsewhere and was not within the scope of this study [18, 19]. Here, we provide evidence that methylation levels can be detected in CTC enriched cell fractions after size-based filtration of blood samples from metastatic breast cancer patients. Overall, at least one gene was methylated in 18/37 patients (48.6%) and four out of 9 genes (*CST6*, *HOXB4*, *ITIH5* and *RASSF1*) were more frequently methylated in the CTC enriched fraction compared to MNCs obtained from healthy individuals.

Following enrichment with a size-based microfilter, 19 patients with >= 1 CTC were identified as CK+/DAPI+/CD45- cells. Out of these 19 CTC positive samples, only 10 samples showed methylation in at least one gene. Overall, we did not find any association between CTC counts and positive methylation. One reason for this finding could be that these CTCs identified as CK+/DAPI+/CD45- had other genes methylated than the ones used in this study or that both techniques identify different subsets of CTCs. Chimonidou et al also observed highly methylated genes in the CTC fraction with an EpCAM-positive/KRT19-negative phenotype [28], indicating the presence of different CTC subpopulations. Since our enrichment technique is independent of epithelial antigen expression, CTCs that have either lost the expression of antigens or have undergone EMT may not be identified by antibody-based enrichment methods and conventional enumeration based on CK expression, but may reveal methylation of tumor associated genes. In fact, a recent study of DNA methylation in single circulating tumor cells indicates tumor-specific epigenetic regulation of EMT-associated genes during blood-born dissemination [37].

CTCs are extremely rare, even in metastatic patients, which challenges and limits their detection and detailed characterization. This limitation was also evident in our study, demonstrating a relatively moderate sensitivity with 18/37 (48.6%) patients positive for at least one gene analyzed. While we used a multigene approach to account for the tumor heterogeneity, the number of samples available for methylation analysis remains limited. Furthermore, pyrosequencing has also a limited sensitivity as shown in this study, and more sensitive quantitative methods are necessary. Clearly, further technical improvements of CTC enrichment are also needed to provide a higher sensitivity allowing for a deeper insight into tumor heterogeneity at the CTC level. Analysis of additional genes could also help to improve the performance of this method.

While methylation of circulating free DNA (cfDNA) from plasma is also possible and perhaps more sensitive, our approach was designed to answer whether CTCs are methylated in breast cancer patients. Moreover, an important limitation of cfDNA is that its origin and more specifically tumor-specific alterations are not known and cannot be linked to specific tumor cell populations [38].

In this study, we used the pyrosequencing technique to assess methylation profiles of size-based enriched CTCs. Compared to the widely used semi-quantitative method MSP, pyrosequencing enables precise and sensitive quantification of DNA methylation [33]. Although highly sensitive, methods like MSP or QMSP are prone to generate false positive results and therefore overestimate the frequency of methylation events [39, 40]. In contrast, pyrosequencing not only facilitates the definition of cut-offs due to quantitative read-outs, but also offers the possibility to control for completeness of bisulfite treatment. This is a clear and essential advantage to many other methylation methods widely used.

Despite efficient CTC enrichment with the present microfilter [18, 19], a variable number of leukocytes still remains on the filter. Consequently, DNA isolated from the filter contains a mixture of both contaminating white blood cells and CTCs. However, except for the DEPArray^Tm^ system [41, 42], no other current CTC enrichment platform allows such pure capture of CTCs, and DEPArray^Tm^ has its own limitations, including long hours to process and small blood volumes. To account for this limitation, we chose candidate genes that are not methylated in leukocytes. We confirmed the absence of methylation by analyzing MNCs of healthy individuals. All 9 genes were confirmed to be unmethylated in 97.8% of control samples consisting of MNCs and methylated to varying extents in five biologically different breast cancer cell lines (data not shown). Furthermore, we applied a stringent cut-off value to define positive methylation using robust quantitative pyrosequencing technology. We also assessed methylation levels using blood from healthy volunteers (n=9) and after filtration of blood samples we found all candidate genes unmethylated (data not shown) in the CTC enriched cell fraction. Together, these data indicate that detected methylation levels result from the CTC enriched fraction on the filter, rather than from specific leukocyte subpopulations, which also remain on the filter.

Finally, we could show in our study that patients with enriched methylated CTCs for the genes *CST6*, *ITIH5* and *RASSF1* had a significantly shorter PFS time compared to patients with unmethylated CTC fractions. In contrast to gene methylation, CTC count did not reveal any prognostic value in our study cohort. Limitations of this study include the small sample size, the heterogeneous breast cancer cohort (e.g. biological subtypes and number of previously received treatments) and the short follow-up time. These limitations could be one reason why CTC counts did not show any prognostic relevance. Clearly, our findings require validation in larger patient cohorts.

In conclusion, we show for the first time that tumor-specific methylation levels can be detected in CTC enriched cell fractions after size-based filtration of blood samples from metastatic breast cancer patients. Because this enrichment platform is independent of cell surface marker expression, capture of heterogeneous CTC subpopulations is possible. Our initial results also provide important evidence of the clinical utility of multigene methylation analysis of enriched CTCs in metastatic breast cancer patients.

## MATERIALS AND METHODS

### Patients and sample collections

A total of 37 patients were enrolled in this prospective proof-of-principle study at the Division of Oncology, Department of Internal Medicine, at the Medical University of Graz. Blood samples were collected from patients with metastatic breast cancer at first diagnosis or at disease progression before starting a new line of systemic treatment. Patients with malignancies other than breast cancer were excluded. Blood samples were also collected from 25 healthy individuals. The study was approved by the local Institutional Review Boards (24-539ex11/12), and all patients and donors gave their written informed consent. For cancer patients, 7.5ml of blood was drawn into CellSave tubes (Veridex LLC, Janssen Diagnostics, Rarities, NJ, USA), and for healthy donors, 9.0ml blood was collected in EDTA tubes (Greiner bio-one, Gremsmünster, Austria). 0.225ml of a 10% neutral-buffered solution containing formaldehyde (4% weight per volume, Sigma-Aldrich, Vienna, Austria) was added into EDTA tubes immediately after blood withdrawal.

Clinical and pathological data for metastatic breast cancer patients were retrieved from clinical records and are presented in Table 3. At the time of the blood draw, the age of the breast cancer patients ranged from 23 to 79 years with a mean of 58±13. The control individuals were aged between 18 and 50 (32±10). Five patients (13.5%) were excluded for prognostic evaluation, because at the time of blood sampling, initial treatment had already started in four cases and one patient did not receive any treatment. Of the remaining 32 patients, disease progression was observed in 18 cases (56.3%) and death in 6 cases (18.8%). Median follow-up time was 338 days±6.3.

**Table 3:** Clinical and pathological characteristics of metastatic breast cancer patients (n=37)

Category	Number	%
Total	37	
**Age (years) at time of sampling**		
Median and range	58 (23-79)	
**Menopausal status**		
Premenopausal	15	40.5
Postmenopausal	21	56.8
Unknown	1	2.7
**Histologic type**		
Invasive ductal/NST	32	86.5
Invasive lobular	3	8.1
Other	2	5.4
**Tumor grade (at primary diagnosis)**		
Grade 1	1	2.7
Grade 2	16	43.2
Grade 3	19	51.4
Unknown	1	2.7
**Primary tumor size**		
pT0/pT1	12	32.4
pT2	10	27.0
pT3/pT4	6	16.2
Unknown	9	24.3
**Lymph node status (at primary diagnosis)**		
N0	13	35.1
N1-3	14	37.8
Unknown	10	27.0
**Estrogen-receptor status (primary tumor)**		
Negative	5	13.5
Positive	32	86.5
**Progesterone-receptor status (primary tumor)**		
Negative	8	21.6
Positive	29	78.4
**HER2 status (primary tumor)**		
Negative	28	75.7
Positive	7	18.9
Unknown	2	5.4
**Subtype (primary tumor)**		
HR+HER2-	25	67.6
HR-HER2-	3	8.1
HER2+	7	18.9
Unknown	2	5.4
**Metastatic location at time of sampling**		
Bone	24	64.9
Lung	15	40.5
Liver	10	27.0
Other locations	23	62.2
**Number of metastatic sites at time of sampling**		
One	16	43.2
Multiple	21	56.8
**Number of previous therapy lines for metastatic disease**		
0	22	59.5
1	7	18.9
>= 2	8	21.6
**Treatment initiated**		
Chemotherapy	14	37.8
Chemotherapy and targeted therapy	9	24.3
Hormone therapy	13	35.1
No treatment	1	2.7

MBC metastatic breast cancer; NST not otherwise specified.

### Cell lines and control samples

The MCF7 breast cancer cells and H1299 lung adenocarcinoma cells were obtained commercially from the American Type Culture Collection (ATCC, Manassas, USA) and cultured according to the supplier's recommendations and were used as control samples. Cell lines were authenticated by DNA short-tandem repeat analysis by the Cell Culture Facility of the Center for Medical Research at the Medical University of Graz (Austria). Fully methylated and unmethylated human control DNA (Zymo Research, Orange, CA, USA) were mixed to obtain following ratios of methylation: 0%, 5%, 10%, 25%, 50%, 75% and 100%.

Mononucleated blood cells (MNCs) were prepared from blood of healthy controls using Ficoll plaque density gradient centrifugation according to the manufacturer's instructions (Lymphoprep, Axis-Shield PoC, Oslo, Norway). MNCs at the interface were harvested and washed in phosphate-buffered saline (PBS). Cells were resuspended in 200μl PBS and stored at -20°C until DNA extraction.

### Spiking experiments

MCF7 cells were counted with the Cellometer Auto 1000 (Nexcelom Bioscience, Lawrence, MA, USA) and the solution was then diluted in PBS to a final concentration of 1 × 10^5^ tumor cells/ml. 50-1000 MCF7 cells were spiked into 7.5ml blood from healthy controls. Spiking of low numbers of MCF7 cells (<100) was performed according to a protocol as previously published [43]. Briefly, 1μl of cell suspension (1 × 10^5^ cells/ml) was transferred to an ultralow attachment 96-well plate (Corning Inc, Corning, NY, USA) and cells were counted under the microscope. Counted cells were then immediately pipetted into 7.5ml of whole blood. The remaining cells in the 96-well plate were also counted and subtracted from the original count in order to more precisely estimate the total number of cells spiked into blood. In detail, the exact numbers for the 100 cell sample were 98, 111 and 88 cells and for the 50 cell samples 36, 62 and 56 cells. Samples were processed for CTC enrichment as described below.

### CTC enumeration

Circulating tumor cells were captured using a size-based microfilter device as previously described [18, 19]. A total of 7.5ml blood was diluted 1:1 with 1x PBS and fixed with a final concentration of 1% formalin (Sigma-Aldrich, Vienna, Austria) for 10 minutes. After fixation, blood was processed through the microfilter at a constant flow rate of 75 ml/hour using a motorized syringe pump. Following filtration, CTC identification and enumeration was done by double immunofluorescence staining including a pan-cytokeratin (CK) antibody and CD45 antibody. Filters containing CTCs were placed onto a glass microscope slide and blocked with blocking buffer consisting of 5% normal goat serum (Life Technologies, Vienna, Austria) and 3% Triton X-100 (Sigma Aldrich) at room temperature for 30 minutes. Next, samples were incubated with primary antibodies, mouse anti-CD45 (Ready-to-use; DAKO, Glostrup, Denmark), and polyclonal rabbit anti-cytokeratin (1:300; DAKO) at room temperature for 1 hour. Samples were then incubated with secondary antibodies, goat anti-mouse Alexa 594 and goat anti-rabbit Alexa 488 (both 1:100, Life Technologies) at room temperature for 1 hour. Finally, samples were counterstained with 4, 6-diamidino-2-phenylindole (DAPI, Invitrogen) and mounted on coverslips with ProLong Gold Antifade mounting media (Life Technologies). The entire area of the microfilter was viewed under a confocal laser-scanning microscope (Zeiss, Oberkochen, Germany), and CTCs were identified as nucleated CK+/DAPI+/CD45- cells.

### DNA extraction

After CTC enumeration, the stained microfilter was removed from the microscope slide by immersion into ice-cold PBS and the filter was then transferred to a QIAGEN Lyse&Spin Basket placed within a 2ml microcentrifuge tube. DNA extraction from captured CTCs and isolated MNCs was performed using the QiampDNA micro Kit (Qiagen, Hilden, Germany) according to the instructions provided in the QIAGEN Lyse&Spin Basket handbook. Briefly, filters were placed into Lyse&Spin Baskets within a 2ml collection tube and 475μl ATL buffer was added. Next, 25μl proteinase K was added and the sample was incubated at 56°C for 1 hour with shaking at 900 rpm. After the incubation period, the tube was centrifuged at 13,000 rpm for 1 min and the flow-through fraction containing the nucleic acids was subjected to DNA isolation using the QIAamp DNA Micro Kit. One μg of carrier RNA was added to each sample and DNA was eluted in 2×25μl elution buffer AE. Genomic DNA from cultured cells was extracted using the QIAmp DNA Mini Kit (Qiagen) according to the manufacturer's instructions. The DNA was dissolved in a final volume of 100μl buffer and quantified using a NanoDrop ND 2000 spectrophotometer (Thermo Fisher Scientific, Bremen, Germany).

### Methylation analyses

For methylation analysis, genomic DNA was subjected to bisulfite conversion using the InnuConvert Bisulfite Basic Kit (Analytik Jena, Jena, Germany) according to the manufacturer's instructions. For CTCs, 40μl (representing the entire extracted genomic DNA) and for cell lines and control samples 1000ng were used for bisulfite conversion. The purified bisulfite-converted DNA was eluted twice in 22μl volume and stored at -20°C for further analysis. DNA methylation analysis of bisulfite-converted DNA samples was performed using pyrosequencing (Pyromark Q24) with PyroMark Q24 Advanced CpG Reagents (Qiagen). The PCR reaction was performed using the PyroMark PCR Kit (Qiagen) according to the protocol with 2μl bisulfite-converted DNA and with an annealing temperature of 56°C and 45 cycles. Final concentration of primers was 0.2μM for all genes. PCR products were visualized on 2% agarose gels to verify amplification of a single band prior to pyrosequencing.

Human MCF-7 breast and H1299 lung cancer cell lines, and DNA from peripheral blood mononuclear cells of healthy individuals were used as methylated and unmethylated controls in each PCR run. Blank controls (without DNA template) were also amplified with each PCR reaction.

For following genes assays were designed using the Pyromark Assay Design Software Version 2 (Qiagen): *AKR1B1, BMP6, CST6, HOXB4, HIST1H3C, ITIH5, NEUROD1, RASSF1, SOX17*. All of these genes have been previously shown to be hypermethylated in breast cancer patients [30, 44]. Primer sequences are listed in Supplementary Table 1. Between five and 15 CpG sites located in the CpG islands of the promoter region were evaluated. All genes have been previously investigated for DNA methylation and primers were positioned to investigate the same or similar CpG sites [26, 30, 31, 44]. All designed assays include a non-CG cytosine in the region for pyrosequencing, as an internal control for complete bisulfite conversion.

Pyrosequencing results were evaluated using the PyroMark Q24 Advanced 3.0.0 software (Qiagen). Methylation data are presented as the percentage of the mean methylation in all CpG sites analyzed per gene. Each pyrogram was assessed for quality controls, including completeness of bisulfite-conversion and expected peak height and sequence. Data were only included in the analyses when all quality controls passed.

A sample was considered as hypermethylated when the mean methylation for the individual gene was higher than the cut-off for the same gene. The cut-off was defined as the mean methylation in the control group (MNCs of healthy controls) plus twice the standard deviation [36]. Additionally, for each individual sample, a cumulative methylation index (CMI) was calculated as the sum of the methylation percentage for the genes analyzed.

### Statistical analysis

Statistical analyses and plotting of data were performed with GraphPad Prism 6.0 (GraphPad Software, San Diego, CA, USA) and SPSS 23 (SPSS, Inc., Chicago, IL, USA). P values < 0.05 were considered significant. The Fisher's exact test was used to test whether differences between methylation positivity of CTCs and MNCs from controls were significant. Overall-survival (OS) was calculated from the date of blood sampling to the date of death. Progression-free survival (PFS) was the interval from the date of blood sampling to the date of clinical progression or death, whichever came first. Univariable survival analyses were performed using Kaplan-Meier plots and the log-rank test for comparisons. Hazard ratios (HR) were calculated for PFS by univariate Cox regression models with 95% confidence interval (CI). Methylation levels from unmethylated samples and low-level methylation samples (5%) were used to calculate Limit of Detection (LOD) for each gene using the formula described by Armbruster et al [34].

## SUPPLEMENTARY MATERIALS FIGURES AND TABLES





## Abbreviations

CTCs: circulating tumor cells
PFS: progression-free survival
EMT: epithelial-mesenchymal transition
EDTA: ethylene-diamine-tetra-acetic acid
MNCs: mononucleated blood cells
CK: pan-cytokeratin
DAPI: 4, 6-diamidino-2-phenylindole
CMI: cumulative methylation index
OS: overall survival
HR: hazard ratios
LOD: limit of detection
CV: coefficient of variation
ER: estrogen receptor
PR: progesterone receptor
HR: hormone receptor
MSP: methylation-specific PCR
QMSP: quantitative methylation-specific PCR
*AKR1B1*: aldo-keto reductase family 1 member B
*BMP6*: bone morphogenetic protein 6
*CST6*: cystatin E/M
*EpCAM*: epithelial cell adhesion molecule
*HER2*: erb-b2 receptor tyrosine kinase 2
*HOXB4*: homeobox B4
*HIST1H3C*: histone cluster 1 H3 family member c
*ITIH5*: inter-alpha-trypsin inhibitor heavy chain family member 5
*KRT19*: keratin 19
*NEUROD1*: neuronal differentiation 1
*RASSF1*: Ras association domain family member 1
*SOX17*: SYR-box 17
