# Supplementary material for: Multigene methylation analysis of enriched circulating tumor cells associates with poor progression-free survival in metastatic breast cancer patients
Source: Oncotarget. 2017 Sep 30;8(54):92483–96. doi: 10.18632/oncotarget.21426 (PMC5696198; doi:10.18632/oncotarget.21426)
Supplement: Supplementary file 1 [file oncotarget-08-92483-s001.pdf]

Multigene methylation analysis of enriched circulating tumor cells associates with poor progression-free survival in metastatic breast cancer patients

SUPPLEMENTARY MATERIALS

Supplementary Table 1: PCR and pyrosequencing primers

Primer name	5' to 3' sequence	PCR product (bp)	# CpGs
AKR1B1 Fw	TGAGTAGGAGAAGGTTTGTTATGGTT	172	13
AKR1B1 Rev	Biotin-CACCCCCAACCCAACCAATCA		
AKR1B1 S	AGTAAGGTGAGGTTTTGG		
BMP6 Fw	GTAAAGGGGTAAATTTTATGGTGGT	393	11
BMP6 Rev	Biotin-CCCTCAATCCTTATCTCTCATAATC		
BMP6 S	TTTATTTAGTAGTTAGGGAGAG		
CST6 Fw	GAGGTAGAATGAGATTAGGGTTAGGT	235	15
CST6 Rev	Biotin-CCCCTCCACCTCCTAAACTCCCTA		
CST6 S	GTTAGTGTAGTAGGAGTTTTTAGA		
HIST1H3C Fw	AGGAGGTTTGTGAGGTTTAT	147	5
HIST1H3C Rev	Biotin-AAAAAAACCAACAAACTTATACCCTT TCCC		
HIST1H3C S	TGAGGTTTATTTGGTGGGATT		
HOXB4 Fw	TGTAAAGTTTTAGGGGTGGGAG	145	8
HOXB4 Rev	Biotin-TCCCTACCCCCAAAACCCCCTACA		
HOXB4 S	GGGTGGGAGGGGGAA		
ITIH5 Fw	TTGAGGGAGAGGAAGAAAGAG	103	5
ITIH5 Rev	Biotin-TTGAGGGAGAGGAAGAAAGAG		
ITIH5 S	GGAGAGGAAGAAAGAGT		
NEUROD1 Fw	AGTAAGGAGTGGGGAGAAGTG	239	12
NEUROD1 Rev	Biotin-CCTAAACAATAATAATCTCATAACCCT AAA		
NEUROD1 S	ATTAATTGGGTTATATAATTTG		
RASSF1 Fw	AGGGAAGGAAGGGTAAGG	268	10
RASSF1 Rev	Biotin-CCAACTCCCCCAACTCAATAAACTCA AACT		
RASSF1 S	GGATTTTGGGGGAGG		
SOX17 Fw	TTTTATAGGGGATATGAAGGTGAAGG	204	15
SOX17 Rev	Biotin-AACTCACCCAACATCTTACT		
SOX17 S	ATATGAAGGTGAAGGG		

Fw forward, Rev Reverse, S Sequencing.

Supplementary Table 2: Summary of methylation results of all nine genes and CTC count in each patient (N=37)

See Supplementary File 1
